# Acute effects of nitrous oxide on visual processing: a connectome study in healthy adults

**DOI:** 10.21203/rs.3.rs-8430361/v1

**Published:** 2026-01-12

**Authors:** Niloufar Pouyan, Chelsea M Kaplan, Tony E Larkin, Eric Ichesco, Maximillian K Eagan, Katrin H Preller, Jeffrey B. Dunworth, Richard E Harris, George A Mashour, Steven E Harte

**Affiliations:** 1. Department of Anesthesiology, University of Michigan Medical School, Ann Arbor, MI, USA; 2. Michigan Psychedelic Center, University of Michigan Medical School, Ann Arbor, MI, USA; 3. Neuroscience Graduate Program, University of Michigan, Ann Arbor, MI, USA; 4. Pharmaco-Neuroimaging and Cognitive-Emotional Processing, Department of Adult Psychiatry and Psychotherapy, Psychiatric University Clinic Zurich and University of Zurich, Zurich, Switzerland; 5. Department of Mathematics, University of Michigan, Ann Arbor, MI, USA; 6. Center for the Study of Complex Systems, University of Michigan, Ann Arbor, MI, USA; 7. Department of Anesthesiology and Perioperative Care, School of Medicine, University of California at Irvine, CA, USA; 8. Susan Samueli Integrative Health Institute, School of Medicine, University of California at Irvine, CA, USA

**Keywords:** Nitrous oxide, Visual processing, Psychedelic state, anterior insula, Network modularity, Hierarchical clustering

## Abstract

**Introduction:**

Nitrous oxide (N_2_O) produces perceptual alterations and changes in large-scale brain network organization, yet its effects on task-specific visual processing remain unclear. Given the prominence of visual alterations during psychedelic exposure, we investigated how subanesthetic N_2_O modulates functional connectivity during visual stimulation.

**Methods:**

Thirteen healthy adults completed a placebo-controlled, crossover fMRI study acquired before and during subanesthetic N_2_O administration (35% in oxygen). Participants viewed a flashing annulus checkerboard and rated evoked subjective visual intensity and unpleasantness. Task-modulated connectivity was assessed using generalized psychophysiological interaction (gPPI) analyses, alongside graph-theoretical measures of modularity and hierarchical clustering to characterize multiscale network organization.

**Results:**

gPPI analyses revealed that increased unpleasantness under N_2_O was associated with reduced connectivity between the right anterior insula (rAI) and clusters in the anterior cingulate cortex (ACC) and right lateral occipital cortex (LOC). Network-level analyses showed redistribution of sensorimotor connectivity toward salience and associative networks, accompanied by reduced modularity and a collapse of hierarchical network organization during visual stimulation under N_2_O.

**Discussion:**

These findings suggest that visual processing under N_2_O is associated with altered salience attribution and increased cross-network communication. Decoupling of rAI from ACC and LOC implicates a mechanism by which affective appraisal of sensory input is modulated, while reduced modularity and hierarchical differentiation indicate diminished stability of canonical functional networks. Together, these preliminary findings indicate that altered visual experience under N_2_O arises from large-scale network reconfiguration and disrupted salience integration rather than changes in early sensory processing.

## Intro

Psychedelics have been informally grouped into classical agents—such as lysergic acid diethylamide (LSD), psilocybin, and DMT—and non-classical agents like ketamine and nitrous oxide (N_2_O)^1^. N_2_O is an odorless, colorless gas widely used in medical and dental settings for its rapid-onset anesthetic, analgesic and anxiolytic effects. At subanesthetic concentrations, N_2_O acts in part through NMDA receptor antagonism and produces similar (but not the same) phenomenological, behavioral, and large-scale neural effects as those induced by classical psychedelics acting through serotonin 5-HT2A receptor agonism^2–5^.

In a resting-state fMRI study in healthy volunteers, we demonstrated that subanesthetic N_2_O induces widespread changes in brain network dynamics, characterized by reduced within-network connectivity and increased between-network integration, particularly within sensory and frontoparietal systems^2^. Another study demonstrated that some N_2_O-induced alterations persisted 24 hours beyond the acute phase, such as in the occipital cortex, suggesting durable effects of N_2_O on sensory processing circuits^6^. Complementing these fMRI findings, EEG data revealed that N_2_O shifts brain oscillatory activity towards less organized and more random network states, with dose-dependent alterations in delta, alpha, and beta frequency bands that reflect diminished efficiency of information processing and integration^4^. Together, these findings suggest that subanesthetic N_2_O, much like classic psychedelics, produces a brain state distinct from natural wakefulness or general anesthesia by disrupting the balance between segregation and integration within brain networks^7,8^. Segregation, one part of this balance, is often quantified by modularity, a measure of the extent to which brain networks are organized into distinct communities^9^.

Visual perception is one of the most prominent and consistent sensory modalities altered by psychedelics, including N_2_O, with reliably reported effects including distortions, intensification of color, and altered salience^10^. Yet, almost all insights into how psychedelics reshape brain dynamics come from resting-state designs, which cannot explain how those network shifts affect the processing of external stimuli^11^. Consequently, the neural mechanisms underlying psychedelic alterations of visual perception remain insufficiently understood.

To address this gap, we employed a structured visual task during fMRI to investigate how subanesthetic N_2_O modulates visual processing and associated brain connectivity in healthy individuals. We combined two complementary analytic approaches. First, we examined task-modulated functional connectivity of the right anterior insula (rAI), a key hub for gating conscious access to sensory information by detecting salient stimuli and orchestrating transations between brain networks essential for awareness^12–14,15^. Importantly, our group has previously demonstrated that rAI responses are sensitive to sensory processing demands during this same visual task, providing task-specific motivation for its selection as an a priori region of interest^16^. Second, we assessed large-scale network modularity and hierarchical clustering to examine changes in the balance between functional segregation and integration, as well as the mesoscale network organization^17,18^. Our aim was to determine whether N_2_O alters neural responses to visual stimulation and to characterize these effects at both regional and systems-level network organization.

## Methods

This investigation was conducted at the University of Michigan Medical School, with ethical oversight and approval granted by the Institutional Review Board (IRB# HUM00096321). All participants were fully briefed regarding potential risks and benefits prior to enrollment and provided written informed consent before study procedures commenced. The analyses presented here are based on data from a registered clinical trial (Clinicaltrials.gov Identifier: NCT03435055), with primary outcomes previously reported^19^.

### Participants

Nineteen healthy adult volunteers were enrolled in this cross-over study. Eligibility criteria included being classified as American Society of Anesthesiologists physical status I and having no history of drug abuse, psychosis, or significant medical or psychiatric conditions. Participants were excluded from analysis if they exhibited excessive head motion (n = 2; see Quality Assurance section) or had incomplete fMRI data (n = 4). Consequently, imaging and behavioral analyses were conducted on data from 13 participants (7 female; mean age ± SD: 24.92 ± 4.03 years).

### Experimental Design and Data Acquisition

Full methodological details—including gas delivery, physiological monitoring, and resting-state acquisition and analysis—are reported in Dai et al. (2023)^2^. The current study focuses exclusively on visual task data, which were not analyzed in previous publications. Briefly, participants underwent a two-visit protocol consisting of a pre-fMRI screening session and a fMRI scanning session within three days. During the screening visit, participants were familiarized with the visual task outside of the scanner. During the fMRI scan session, participants completed the visual task before and after administration of subanesthetic N_2_O (35% in oxygen), delivered via facemask after a baseline period under placebo (medical air).

N_2_O was administered using MRI-compatible anesthesia equipment under anesthesiologist supervision. Standard pre-scan tolerance checks and physiological monitoring (electrocardiogram, blood pressure, pulse oximetry, capnography) were used throughout. Prophylactic and rescue medications were available for anticipated side effects. Earplugs and headphones minimized environmental noise for the participants during scanning.

All imaging was performed on a 3T Philips Achieva scanner (Best, Netherlands) at the University of Michigan Medical Center. Whole-brain functional images were obtained using a T2*-weighted echo-planar imaging sequence with the following parameters: 48 axial slices, repetition time = 2000 ms, echo time = 30 ms, slice thickness = 3 mm, field of view = 200 × 200 mm, and flip angle = 90°. Each visual functional scan lasted 200 seconds. High-resolution anatomical images were acquired to facilitate co-registration with functional data.

### Visual Task

**Figure 1.**
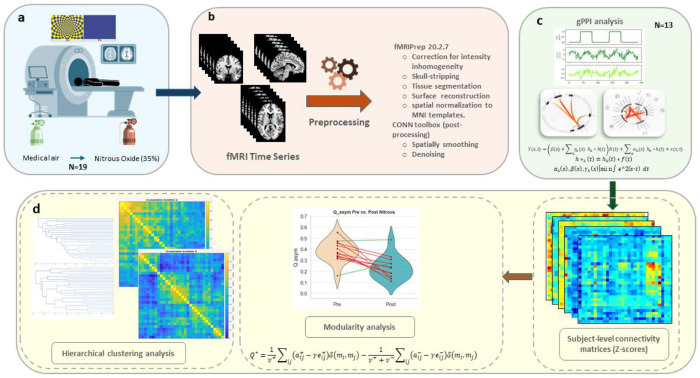
Schematic of the experimental design and analysis pipeline. **a)** Participants completed a crossover fMRI protocol under placebo (medical air) and subanesthetic nitrous oxide while performing a visual task, which was a dynamic visual stimulus consisting of a 3-minute movie clip featuring alternating 20-second blocks of an 8 Hz flashing blue-yellow annulus checkerboard (left panel) and a static fixation cross (right panel). Figure reproduced from Harte et al. (2016). **b)** fMRI time series underwent standard quality control, preprocessing, and postprocessing procedures prior to analysis. **c)** Preprocessed fMRI data were analyzed using generalized psychophysiological interaction (gPPI) models. **d)** The resulting ROI-to-ROI gPPI connectivity estimates were assembled into subject-level connectivity matrices (Fisher z-transformed), which were subsequently examined using graph-theoretical modularity analysis and hierarchical clustering to characterize large-scale and mesoscale network reorganization.

The visual task consisted of a 3-minute dynamic visual stimulation protocol administered using E-Prime 2.0^20^, as previously described ^16,21^ Participants wore MRI prism glasses and viewed a high-resolution LED monitor displaying a blue and yellow annulus checkerboard flashing at 8 Hz and fixed at an intensity of 76 lux, alternating every 20 seconds with a static fixation cross on a solid background (figure 1a). Participants were instructed to keep their eyes open and maintain focus on the visual stimulus. After each run of the task, participants rated the perceived intensity and unpleasantness evoked by the visual stimulus using a 0–10 numerical scale, with 10 representing the most intense or most unpleasant experience imaginable.

### fMRI Preprocessing

Structural and functional MRI data were preprocessed using *fMRIPrep* 20.2.7 in a BIDS-compliant workflow (Figure 1b)^22^. Standard preprocessing included correction for intensity inhomogeneity, skull-stripping, tissue segmentation, surface reconstruction, and spatial normalization to MNI templates. BOLD images were corrected for head motion and slice timing, co-registered to structural images, and resampled to both native and standard space. Confound regressors were estimated (motion, CompCor, global signals, framewise displacement, and DVARS) and carried forward to first-level analysis.

All preprocessed data were subsequently imported into the CONN toolbox^23^, where functional images were spatially smoothed (6 mm FWHM) and denoised using the default pipeline (aCompCor, rigid motion regressors, motion outlier scans via ART toolbox, task/session effects, linear trends). A high-pass filter of 0.008 Hz was applied. Details of each preprocessing step are provided in the Supplementary Material.

### Quality Assurance

All preprocessed scans underwent rigorous quality assessment to ensure suitability for further analysis. Visual inspection of the *fMRIPrep*-generated HTML reports was conducted to identify anatomical or functional anomalies (e.g., poor skull stripping, misalignment, failed normalization). For denoising, six head motion parameters and their first temporal derivatives were examined across all subjects. Two participants were excluded if they exhibited excessive motion, defined as exceeding three interquartile ranges (IQR) above the group median in any motion parameter^24^. No other participants were excluded on the basis of preprocessing or anatomical abnormalities.

### Functional Connectivity and Network Modularity Analysis

To characterize N_2_O –induced alterations in functional brain organization during visual stimulation, we employed a two-level analysis approach. First, we used generalized psychophysiological interaction (gPPI) models to examine condition-dependent changes in functional connectivity during the visual task^25^. Second, we derived graph-theoretical modularity metrics from the resulting connectivity matrices to assess systems-level network reorganization.

### Generalized Psychophysiological Interaction (gPPI) Analysis

Psychophysiological interaction analyses were conducted to assess task-modulated changes in connectivity between the pre- and post- N_2_O administration processing of the visual task (Figure 1c)^25^. Seed regions included a total of 32 regions of interest (ROIs), which were selected from the CONN toolbox’s default network atlas. These ROIs correspond to nodes spanning eight canonical large-scale functional brain networks: the default mode (DMN), salience (SN), dorsal attention (DAN), sensorimotor (SMN), visual (VN), frontoparietal (FPN), language (LN), and cerebellar networks (CN). The atlas was derived using hierarchical clustering complete-linkage with Sørensen distances^26^, optimal leaf ordering,^27^ applied to resting-state fMRI data from the Cambridge 1000 Connectomes Project (n = 198), preprocessed and denoised using CONN’s default pipeline (http://www.nitrc.org/projects/fcon_1000) ^28^.

For each pair of seeds, a generalized PPI model was constructed with the seed region’s BOLD signal as the physiological regressor, task-specific boxcar functions as psychological regressors (convolved with the canonical hemodynamic response function in SPM), and their interaction terms as the psychophysiological regressors. Connectivity modulation across task conditions was indexed by the regression coefficients of the interaction terms. ROI-to-ROI correlation matrices were computed and Fisher z-transformed to improve normality, then analyzed using CONN’s Functional Network Connectivity framework with multivariate parametric statistics.

### Group-Level Analyses and Statistical Inference

Second-level (group) analyses were conducted using both ROI-to-ROI and seed-to-voxel approaches. The seed-to-voxel analysis assesses connectivity between a given seed ROI and all other voxels across the whole brain. For the seed-to-voxel gPPI analyses, we focused on the right anterior insula (rAI), selected *a priori* for its established role in gating conscious access to sensory information^12–15^. The rAI seed corresponded to the CONN network atlas ROI Salience network: Anterior Insula [R] (MNI: 47, 14, 0). Group-level voxel wise statistics were thresholded at a voxel-level cluster-forming threshold of *p* < 0.001 (uncorrected) with a cluster-level family-wise error correction of *p*FWE < 0.05, as implemented via Gaussian Random Field theory in CONN.

Complementing the seed-to-voxel analyses, ROI-to-ROI analyses were performed to examine, for each of the 32 atlas-defined regions, how its functional connectivity with all other regions changed across conditions (N_2_O versus medical air placebo). Statistical inference followed CONN’s default two-stage cluster-based thresholding procedure: first, individual connections (edges) were thresholded at an uncorrected p < 0.01; second, clusters of suprathreshold connections were evaluated for significance using FWE correction at p < 0.05. The specific correction level depended on the level of inference within the atlas framework: network-level inference used network-based FWE correction (mass and intensity) based on CONN’s hierarchical clustering of functionally and anatomically similar regions, whereas ROI-level inference used FWE correction applied to all connections associated with a given atlas ROI (mass and intensity).

In all second-level models, we examined the primary contrast of N_2_O versus placebo. In addition, subjective ratings of visual task intensity and unpleasantness were included as covariates to examine the relationship between individual sensory experiences and functional connectivity changes.

### Network Modularity Analysis

To assess global systems-level alterations in brain organization under N_2_O, we computed modularity from the gPPI-derived ROI-to-ROI connectivity matrices obtained in the prior analysis (Figure 1d). These matrices encode directed, weighted connections modulated by task condition, containing both positive and negative weights (~40% negative edges in our dataset). Given these characteristics, we employed the Louvain community detection algorithm with the *negative_asym* setting from the Brain Connectivity Toolbox^17,29^.This variant accommodates asymmetric networks with mixed edge polarity, enabling positive and negative interactions to inform community structure without conflating their influence. The asymmetric measure of modularity Q* can be formally defined as^17^:

$$Q^{*}=\frac{1}{v^{+}}\sum_{ij}\left(a_{ij}^{+}-\gamma e_{ij}^{+}\right)\delta \left(m_{i},m_{j}\right)-\frac{1}{v^{+}+v^{-}}\sum_{ij}\left(a_{ij}^{-}-\gamma e_{ij}^{-}\right)\delta \left(m_{i},m_{j}\right)$$

where *v*^+^ and *v*^−^ are the total sum of positive and negative edge weights, respectively; $a_{ij}^{+}$ and $a_{ij}^{-}$ represent the positive and negative edge weights between nodes *i* and *j*; *γ* is the structural resolution parameter; $e_{ij}^{+}$ and $e_{ij}^{-}$ are the expected positive and negative edge weights between nodes *i* and *j* under a specified null model and *δ*(*m_i_*, *m_j_*) is the Kronecker delta function, equal to 1 if *m_i_* = *m_j_* and 0 otherwise.

Although directionality of gPPI-derived connectivity remains a topic of debate, we employed the *negative_asym* function to preserve potential asymmetries in task-modulated connectivity, allowing both positive and negative influences to be appropriately represented in the modularity analysis^30^. The resulting modularity index (Q*) quantifies the extent to which the brain’s functional architecture is segregated into distinct modules characterized by dense within-module and sparse between-module connections. Lower modularity values (Q*) indicate reduced functional segregation and greater cross-network integration, whereas higher Q* values reflect stronger segregation into distinct modules with dense within-module and sparse between-module connectivity^31^.

### Hierarchical clustering Analysis

To characterize the hierarchical organization of task-modulated functional connectivity, agglomerative hierarchical clustering was applied to each participant’s z-transformed gPPI-derived ROI-to-ROI connectivity matrix (Figure 1d). For each subject, the connectivity matrix was converted to a distance matrix defined as:

$$D_{ij}=1-r_{ij,}$$

where *r_ij_* denotes the gPPI connectivity estimate between regions *i* and *j*. This transformation maps stronger positive connectivity to smaller distances and weaker or negative connectivity to larger distances, rendering the matrix suitable for hierarchical clustering. Subject-level clustering was performed using MATLAB’s average linkage method, yielding an individual dendrogram for each participant.

Because consensus clustering and hierarchical analyses require specification of a mesoscale resolution, we selected a primary cluster number for group-level analyses. A resolution of *k* = 8 was chosen based on biological plausibility and alignment with commonly reported large-scale functional networks, as well as the network granularity of the atlas used in this study^32–34^. To assess the robustness of this choice, we evaluated a range of candidate resolutions using multiple internal validity metrics (silhouette coefficient, Davies–Bouldin index, Calinski–Harabasz criterion, and gap statistic^35–38^). These measures did not converge on a single consensus optimum, instead revealing local optima at both coarse (*k* = 2) and finer (*k* ≥ 8) scales, consistent with the multiscale organization of brain networks. Importantly, the overall pattern of results was stable across resolutions, indicating that the reported findings are not driven by a specific choice of *k*. Coarser-resolution solutions, including an optimal *k* = 2 partition capturing broad network segregation, are presented in the Supplementary Material (Figure s5).

To identify stable group-level structure within each condition, subject-level partitions were aggregated into condition-specific co-association matrices. For each condition, the co-association value *P_ij_* was defined as the proportion of subjects for whom ROIs *i* and *j* were assigned to the same cluster:

$$P_{ij}=\frac{1}{S}\sum_{s=1}^{s}\delta \left(m_{i}^{\left(s\right)},m_{j}^{\left(s\right)}\right)$$

where *S* denotes the number of subjects and $m_{i}^{\left(s\right)}$ is the cluster assignment of ROI *i* for subject *s*. *δ* (*m_i_, m_j_*) is the Kronecker delta function, equal to 1 if *m_i_ = m_j_* and 0 otherwise. The resulting 32×32 co-association matrix, with values ranging from 0 to 1, reflects the stability of pairwise ROI co-clustering across participants within a given condition.

Each condition-level co-association matrix was converted into a consensus distance matrix:

$$D_{ij}^{\left(\text{cons}\right)}=1-P_{ij},$$

and subjected to agglomerative hierarchical clustering using average linkage. This procedure yielded a condition-specific consensus dendrogram, capturing the hierarchical organization of stable ROI groupings across subjects. Consensus partitions were obtained by cutting the dendrogram at a fixed number of clusters (*k*), selected as described below.

To assess the extent to which N_2_O altered group-level mesoscale organization, we directly compared the condition-specific co-association matrices. The upper-triangular (non-redundant) elements of each matrix were vectorized and compared using Pearson correlation, providing a continuous, model-free measure of similarity in ROI co-clustering structure between the placebo and N_2_O conditions. Higher correlations indicate more similar hierarchical organization, whereas lower correlations reflect greater reconfiguration of mesoscale functional structure.

## Results

### Subjective Ratings of the Visual Task

Subjective ratings of visual stimulus-evoked intensity and unpleasantness were obtained following each run of the visual task under both placebo and N_2_O conditions. Change scores (post–pre) were calculated for each measure. The mean change in intensity (Δ Intensity) was 0.54 (SD = 2.47), while the mean change in unpleasantness (Δ Unpleasantness) was −0.58 (SD = 1.78). Normality testing using Shapiro–Wilk (p < 0.05) revealed non-normal distributions. Thus, Wilcoxon signed-rank tests were used for pre–post comparisons. No significant differences were found for either construct. Despite the absence of group-level effects, inter-individual variability was pronounced: some participants reported increases in perceived intensity or unpleasantness under N_2_O, whereas others experienced reductions (Figure S1, Supplementary Material).

### gPPI Seed-to-Voxel Connectivity of the Right Anterior Insula (rAI)

**Figure 2.**
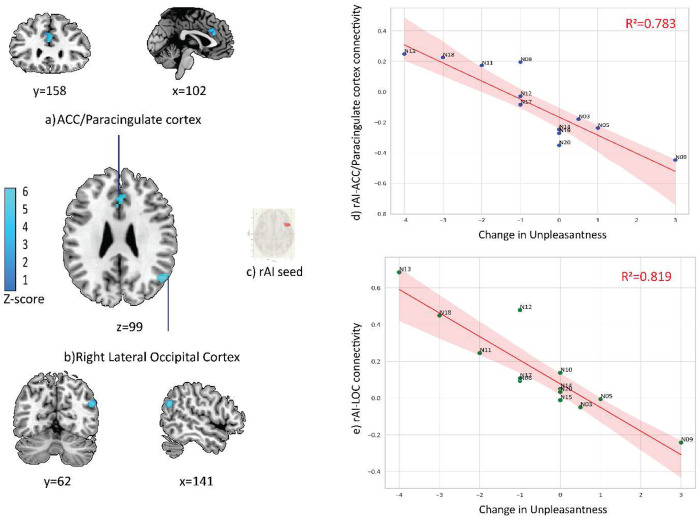
Subjective ratings of visual stimulus-evoked unpleasantness change were associated with decreased connectivity between the right anterior insula (rAI) and two significant clusters: one overlapping the **a)** anterior cingulate cortex (ACC) and bilateral paracingulate cortices (MNI: +00, +30, +24; 101 voxels; cluster pFWE = 0.0048), and **b)** another overlapping the superior division of the right lateral occipital cortex (LOC; MNI: +50, −64, +26; 79 voxels; cluster pFWE = 0.0194). **c)** rAI seed used for the analysis. **d)**Association between ACC connectivity (Z-scores) and change in unpleasantness ratings. **e)** Association between LOC connectivity (Z-scores) and change in unpleasantness ratings. For plots d and e, each point represents a subject. The regression line and shaded 95% confidence interval are shown, with subject number and R^2^ values annotated on the plots.

Using the rAI as an *a priori* seed, we investigated N_2_O -related alternations in whole-brain functional connectivity. The main effect of N_2_O on rAI connectivity was not significant when considered in isolation. However, including subjective ratings of perceived changes in intensity and unpleasantness under N_2_O revealed distinct response patterns: intensity change scores did not significantly relate to rAI connectivity, whereas unpleasantness change scores had significant negative associations with altered connectivity patterns (random field theory parametric statistics, voxel threshold *p* < 0.001 uncorrected, cluster-size FWE-corrected *p* < 0.05). Two significant clusters emerged (Figure 2). The first cluster (MNI coordinates: +00, +30, +24; cluster size = 101 voxels; size p-FWE = 0.0048) overlapped primarily with the anterior cingulate cortex (ACC) and bilateral paracingulate gyrus. The second cluster (MNI coordinates: +50, −64, +26; cluster size = 79 voxels; size p-FWE = 0.0194) overlapped predominantly with the superior division of the right lateral occipital cortex (rLOC).

### gPPI ROI-to-ROI, Network Modularity, and Hierarchical Analyses:

#### ROI-Level Inference

**Figure 3.**
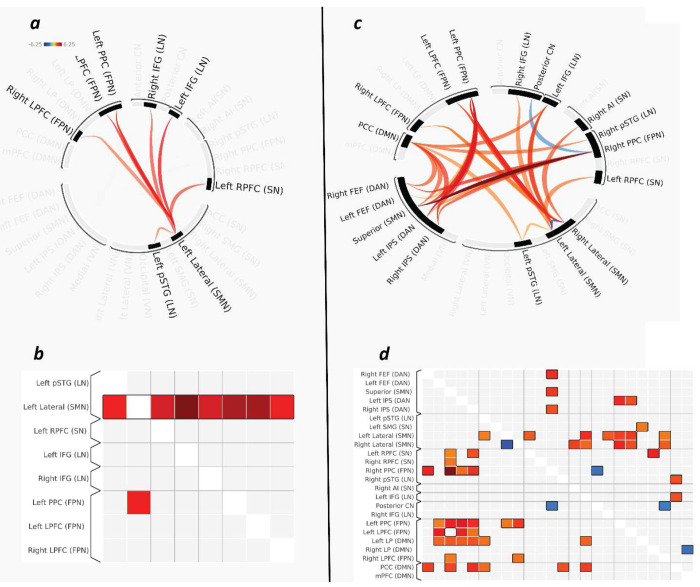
Task-modulated functional connectivity results. **(a,b)** ROI-level generalized psychophysiological interaction (gPPI) results shown as a ring plot **(a)** and connectivity matrix **(b)**, demonstrating that the left lateral sensorimotor cortex exhibits a significant collective change in task-dependent connectivity with regions belonging to the language, salience, and frontoparietal control networks (cluster-mass statistic, p_FWE_< 0.05). **(c,d)** Network-level gPPI results displayed as a ring plot **(c)** and connectivity matrix **(d)**, revealing a significant pattern of task-modulated connectivity across large-scale functional systems (network-level mass statistic = 832.57, p_FWE_= 0.001). Abbreviations IFG, inferior frontal gyrus; pSTG, posterior superior temporal gyrus; LP, latera parietal cortex; LPFC, lateral prefrontal cortex; MPFC, medial prefrontal cortex PPC, posterior parietal cortex; RPFC, rostral prefrontal cortex; AI, anterior insula; ACC, anterior cingulate cortex; SMG, supramarginal gyrus; FEF, frontal eye fields; IPS, intraparietal sulcus; PCC, posterior cingulate cortex; CN, cerebellar network; LN, language network; SN, salience network; FPN, frontoparietal control network; DMN, default mode network; DAN, dorsal attention network; SMN, sensorimotor network.

Using family-wise error correction at the ROI level (ROI- level mass statistic = 95.43, pFWE = 0.024), the left lateral sensorimotor cortex of the SMN showed significant task-dependent connectivity changes with a distributed cluster of regions spanning the FPN, LN, and SN networks (Figure 3a and b).

#### Network-Level Inference

At the network level, a significant cluster of modulated connections was identified (network-level mass statistic= 832.57, p_FWE_ = 0.001). This effect spanned multiple large-scale networks, including FPN, SMN, DAN, DMN, LN, and SN networks, indicating a reorganization of global network topology. Inference was based strictly on the network-level cluster— individual edges within this set were not interpreted as significant unless they survived correction independently. Only one connection—between the superior sensorimotor cortex and the right frontoparietal posterior parietal cortex seeds (atlas seeds)—reached edge-level significance (Figure 3 c and d).

#### Network Modularity Analysis

**Figure 4.**
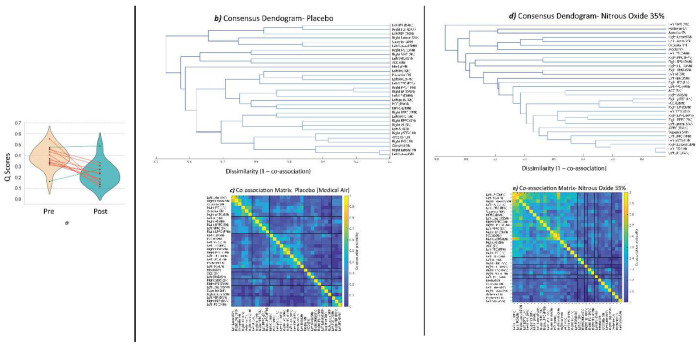
Task-modulated functional network organization under placebo and nitrous oxide. (a) Changes in network modularity (Q) during the visual task pre- and post–nitrous oxide administration. Violin plots depict modularity values (Q) derived from gPPI connectivity matrices using Louvain community detection. Red lines represent participants exhibiting decreases in modularity, reflecting greater network integration, while green lines represent increases. Group-level analysis demonstrated a significant reduction in Q following nitrous oxide administration (ΔQ = −0.15, SD = 0.14; t(12) = −3.87, p = 0.002), consistent with reduced functional segregation during visual processing. (b,c) ROI-level co-association matrices summarizing the proportion of subjects in whom each pair of regions was assigned to the same cluster, shown for the placebo (b) and nitrous oxide (c) conditions. (d,e) Consensus hierarchical dendrograms derived from the co-association matrices, illustrating mesoscale network organization under placebo (d) and nitrous oxide (e) Abbreviations: Q, modularity; gPPI, generalized psychophysiological interaction; ROI, region of interest; IFG, inferior frontal gyrus; pSTG, posterior superior temporal gyrus; LP, latera parietal cortex; LPFC, lateral prefrontal cortex; MPFC, medial prefrontal cortex PPC, posterior parietal cortex; RPFC, rostral prefrontal cortex; AI, anterior insula; ACC, anterior cingulate cortex; SMG, supramarginal gyrus; FEF, frontal eye fields; IPS, intraparietal sulcus; PCC, posterior cingulate cortex; CN, cerebellar network; LN, language network; SN, salience network; FPN, frontoparietal control network; DMN, default mode network; DAN, dorsal attention network; SMN, sensorimotor network.

To further characterize large-scale network reorganization, we computed network modularity (Q*) from the gPPI-derived connectivity matrices. Following N_2_O administration, Q* was significantly reduced compared with pre-administration values (ΔQ* = −0.15, SD = 0.14; t(12) = −3.87, p = 0.002) indicating a shift toward greater network integration and reduced functional segregation during visual processing (Figure 4a). This pattern of decreased modularity was consistent across nearly all participants, with only two exceptions, reflecting a moderate but robust reconfiguration of brain network architecture. The observed reduction in modularity suggests that N_2_O facilitates a more integrated network state, whereby communication between distinct functional modules is less constrained. The change in modularity scores (ΔQ*) showed no significant correlation with changes in unpleasantness or intensity ratings (Supplementary Material, Figure S2).

#### Hierarchical Clustering Analysis

Hierarchical clustering of consensus co-association matrices revealed pronounced condition-dependent differences in the organization of task-modulated functional connectivity (Figure 4b–e). In the placebo condition, the dendrogram exhibited a well-stratified hierarchical structure, characterized by merging of regions belonging to the same canonical functional systems early along the dendrogram hierarchy. Visual regions clustered at low dissimilarity values, followed by the emergence of coherent salience, language, default-mode, and frontoparietal subtrees that remained segregated until higher hierarchical levels (Figure 4b). As dissimilarity increased, these subnetworks merged in an orderly manner, yielding a hierarchical organization in which functionally specialized systems were preserved at lower levels and progressively integrated at higher levels.

This hierarchical structure was supported by the corresponding co-association matrix (Figure 4c), which showed strong within-network co-assignment among VN, SN, DMN, and SMN, alongside weaker co-assignment between networks. Notably, although the clustering procedure was agnostic to canonical resting-state network labels, the resulting consensus clusters closely resembled established large-scale functional systems, indicating that canonical functional network organization was preserved during task performance under placebo.

In contrast, the dendrogram obtained under N_2_O displayed a markedly altered organization characterized by a compressed, cascade-like structure (Figure 4d). Regions merged across a narrow range of dissimilarity values, with reduced formation of distinct subtrees corresponding to canonical networks. Consistent with this pattern, the co-association matrix under N_2_O (Figure 4e) showed reduced within-network cohesion and increased cross-network co-assignment, indicating diminished modular stability. Visual, salience, language, sensorimotor, and default-mode regions failed to form stable, isolated clusters and instead accreted sequentially into mixed communities. Together, these findings indicate a flattening of hierarchical network organization and increased inter-network mixing under N_2_O.

## Discussion

We sought to elucidate the neural mechanisms underlying visual processing under subanesthetic N_2_O by integrating targeted and whole-brain approaches to interrogating functional connectivity.

### Nitrous Oxide Modulates rAI–ACC and rAI–LOC Connectivity with Subjective Unpleasantness

Although group-level analyses of subjective reports did not reveal significant changes in perceived intensity or unpleasantness of the visual stimulus following N_2_O administration, notable interindividual differences emerged in response to visual stimulation^39^. Some participants experienced heightened visual-evoked unpleasantness and intensity, whereas others reported no change or even a reduction. This marked variability is particularly noteworthy given N_2_O’s widespread use as an analgesic^40^. On the neural level, N_2_O altered rAI connectivity in a manner consistent with changes in subjective unpleasantness, but not intensity. Specifically, participants who reported increased unpleasantness under N_2_O showed reduced task-related coupling between the rAI and two clusters in the ACC/paracingulate cortex and the right LOC. This decoupling could provide several insights into how N_2_O modulates salience processing and sensory-affective integration during visual stimulation.

The rAI and ACC are canonical hubs of the salience network, implicated in detecting behaviorally relevant stimuli and coordinating dynamic switching between sensory and higher-order networks^41,42^. Reduced rAI–ACC coupling as a function of increased unpleasantness suggests a disruption of this integrative mechanism. When connectivity between these hubs is weakened, the normal coordination of salience detection and executive resources may be impaired, potentially leading to maladaptive appraisal of sensory input^43^. This aligns with evidence from pain and affective neuroscience showing that insula–ACC connectivity is critical for the attribution of affective valence to sensory experiences^44–47^. In this context, N_2_O may dysregulate the affective “gating” of visual input, increasing the likelihood that certain visual stimuli (e.g., flashing checkerboards) will be perceived as unpleasant by some individuals^16^.

The observed decoupling with the right LOC further supports this interpretation. The LOC is central to higher-order visual processing and object representation^30,48^. During exposure to N_2_O, reduced coupling between the rAI and LOC in participants who experienced greater unpleasantness suggests that the integration of salience signals with visual cortical representations was attenuated. This could mean that sensory input was processed in a manner that is less constrained by salience-driven modulation. Such a mechanism would be consistent with reports of psychedelic-induced visual distortions, where perceptual content is experienced with altered salience or affective tone^49^.

Taken together, the decoupling of rAI to ACC and LOC suggests that N_2_O’s modulation of subjective unpleasantness reflects a reconfiguration of how salience and affective networks interact with cortical visual processes. In other words, unpleasantness may arise when the insula’s normal role in orchestrating network transitions is compromised by N_2_O, disrupting the balance between bottom-up sensory input and top-down appraisal^13^.

### Nitrous Oxide Induces Large-Scale Network Reorganization and Reduced Modularity

Considering the altered connectivity across ROIs, our results show that visual processing under N_2_O shifts sensorimotor connectivity toward greater integration with higher-order evaluative and salience networks. Specifically, the left lateral sensorimotor cortex showed increased coupling with regions implicated in executive control, salience detection, and associative processing, while connectivity with the contralateral sensorimotor cortex decreased. In the context of the visual task, the redistribution of sensorimotor connectivity toward salience and control networks may indicate that circuits typically devoted to modality-specific processing were instead recruited into broader evaluative systems implicated in monitoring, contextualizing, and assigning salience to incoming sensory input. The absence of strong involvement of early visual cortices in the network-level cluster further supports the idea that N_2_O’s impact during visual processing occurs primarily at the level of large-scale integrative systems, rather than at the level of sensory encoding per se. Stronger coupling with salience and frontoparietal networks may facilitate heightened aversive appraisal of sensory input as observed in individuals with chronic widespread pain^50–53^. Moreover, increased connectivity with language-related regions might point to the functional recruitment of language, association, and interpretation processes to help understand, label, and potentially regulate aversive sensory experiences^54^.

At the network level, we observed a significant cluster of modulated connections using network-level mass and intensity statistics, reflecting robust reorganization of functional connectivity across large-scale systems (Figure 4). This cluster spanned multiple resting-state networks, including the FPN, SMN, LN, DAN, and DMN. Notably, one connection—between the superior sensorimotor cortex and the right posterior parietal node of the FPN—reached edge-level significance, suggesting it may play a pivotal role in the broader reorganization. Interestingly, despite the visual nature of the task, the visual network did not appear in the significant cluster, indicating that changes in sensory processing under N_2_O might be at the level of higher-order networks rather than early visual regions.

Graph-theoretical analysis revealed a significant decrease in modularity under N_2_O, indicating that large-scale brain networks became more integrated and less segregated. This finding is consistent with EEG evidence from Kim et al. ^55^, who reported that N_2_O alters network topology, including modularity, in ways that disrupt the balance between information integration and segregation. Importantly, our hierarchical and consensus clustering analyses extend this result by showing that reduced modularity under N_2_O is accompanied by a collapse of hierarchical network organization, marked by diminished stability of mesoscale communities and increased inter-network mixing. Rather than reflecting a uniform increase in connectivity, this pattern suggests a reconfiguration in which canonical functional systems lose their hierarchical separation. Reduced modularity and hierarchical depth together reflect less constrained communication between functional modules, a network state shown by Godwin et al. ^56^ to facilitate cross-network interactions associated with altered perceptual experiences.

Taken together, these multiscale findings—from localized ROI-level changes to distributed network-level effects and global reductions in modularity—highlight a hierarchical reorganization of brain connectivity induced by N_2_O. This reconfiguration is consistent with the acute effects of N_2_O, including altered sensory processing and salience attribution. More broadly, our results support the view that psychedelic compounds exert their influence by redistributing connectivity from locally segregated, unimodal systems toward more transmodal, integrative networks, thereby reshaping the functional architecture that supports altered conscious experience^7,57^.

### Limitations

This work has important limitations. First, this work represents an exploratory, preliminary investigation into the effects of N_2_O on visual processing. The sample size was relatively small, which limits statistical power and the generalizability of the findings. While the observed effects were robust at multiple scales, replication in larger samples and with different atlases is needed to confirm the reliability of these network-level and behavioral patterns.

This study was conducted in healthy volunteers and the generalizability to clinical populations is unknown. Although N_2_O shares some phenomenological and neurobiological features with classical psychedelics, there are also marked differences ^5^. Thus, findings from N_2_O studies should be interpreted with caution when generalizing to other psychedelics, particularly regarding network-level effects, subjective experiences, and therapeutic mechanisms.

## Conclusion

Taken together, these behavioral and neuroimaging findings suggest that N_2_O modulates salience processing and cross-network communication during visual perception. Specifically, N_2_O reshapes functional connectivity from locally segregated, unimodal networks toward more transmodal, integrative systems. This shift is reflected globally by reduced network modularity and, at the mesoscale, by a collapse of hierarchical organization revealed through consensus and hierarchical clustering, indicating diminished stability of canonical functional networks and increased inter-network mixing. At the regional level, reduced coupling between the rAI, ACC, and LOC is associated with subjective unpleasantness, while redistribution of sensorimotor connectivity further reflects a broader reorganization of functional architecture. Together, these multiscale alterations provide preliminary evidence that N_2_O alters visual experience through higher-order integrative and salience-related network dynamics, rather than early visual processing.

## Supplementary Material

This is a list of supplementary files associated with this preprint. Click to download.

• Supplementarymaterial.docx
